# Does Gender Matter in the Relationship between Anxiety and Decision-Making?

**DOI:** 10.3389/fpsyg.2017.02231

**Published:** 2017-12-19

**Authors:** Fenghua Zhang, Leifeng Xiao, Ruolei Gu

**Affiliations:** ^1^School of Psychology, Jiangxi Normal University, Nanchang, China; ^2^Key Laboratory of Psychology and Cognition Science of Jiangxi, Jiangxi Normal University, Nanchang, China; ^3^Collaborative Innovation Center of Assessment toward Basic Education Quality, Beijing Normal University, Beijing, China; ^4^CAS Key Laboratory of Behavioral Science, Institute of Psychology, Chinese Academy of Sciences, Beijing, China; ^5^Department of Psychology, University of Chinese Academy of Sciences, Beijing, China

**Keywords:** decision-making, anxiety, gender difference, Iowa Gambling Task (IGT), Game of Dice Task (GDT)

## Abstract

There is an ongoing debate about whether and how anxiety level affects behavioral performance in risk and/or ambiguous decision-making. According to the literature, we suggest that gender difference might be a confounding factor that has contributed to heterogeneous findings in previous studies. To examine this idea, 135 students who participated in this study were divided into six groups according to their gender (male/female) and trait anxiety level (high/medium/low; measured by the Trait form of Spielberger’s State-Trait Anxiety Inventory). All groups finished the Iowa Gambling Task (IGT) for ambiguous decision-making, and the Game of Dice Task (GDT) for risk decision-making. Behavioral results revealed that the IGT but not the GDT showed an interaction between anxiety and gender. Specifically, men outperformed women in the IGT, but only when their trait anxiety levels were low. Meanwhile, the GDT showed a main effect of anxiety grouping, such that low anxious participants were more risk-seeking than their medium anxious counterparts. These findings indicate that gender selectively modulates the influence of anxiety on ambiguous decision-making, but not risk decision-making. The theoretical and practical implications of the current findings are discussed.

## Introduction

Anxiety could be defined as “an unpleasant emotional state or condition that is characterized by subjective feelings of tension, apprehension, and worry, and by activation or arousal of the autonomous system” (Schwarzer et al., 1987; as cited by Bekker et al., 2003, p. 255). The influence of anxiety on daily life is multifaceted and may manifest on the physiological, cognitive, and behavioral levels. Regarding the physiological aspect, high anxious individuals may experience increased heart rate, respiration, sweating, and muscular tension (Grecucci et al., 2012). Regarding the cognitive aspect, high anxious individuals are more likely to attend to uncertain threat-related stimuli, and show difficulties in attentional disengagement (Bishop et al., 2004; Koster et al., 2006; Bishop, 2009). These changes may in turn affect behavioral performance. The current study focuses on the behavioral level, that is, the influence of anxiety on decision-making.

Decision-making is a complex phenomenon in which various cognitive processes are involved, and could be categorized in many ways. In an uncertain environment (i.e., the relationship between options and outcomes is probabilistic), decision-making could be divided into two types according to whether outcome probabilities are explicitly revealed, that is decision-making under risk and that under ambiguity (Lipshitz and Strauss, 1997; Bach et al., 2009). Decision-making under risk means that when facing different options, the exact probability of each kind of possible outcome is knowable to people, such as Russian roulette; in contrast, decision-making under ambiguity refers to the situations that this probability is unknown, such as terrorist attack and natural disasters (Knight, 1921; Ellsberg, 1961; Camerer and Weber, 1992; Huettel et al., 2006; Liu and Colman, 2009). Many studies have argued that anxiety affects both risk decision-making and ambiguous decision-making, but their results are heterogeneous (Hartley and Phelps, 2012; Paulus and Yu, 2012).

Regarding risk decision-making, Eisenberg et al. (1995) first reported that participants with high level of anxiety showed a risk-avoidant tendency, that is, they preferred low-risk over high-risk options when outcome probabilities were held constant. Follow-up research supports the reliability of this finding (e.g., Raghunathan and Pham, 1999; Grecucci et al., 2012). To explain this phenomenon, Hartley and Phelps (2012) suggest that high anxious individuals show an attentional bias toward potential adverse outcomes of risky decisions. However, Mitte (2007) found that the relationship between anxiety and risk avoidance was only significant when the risk level was described in verbal format, but not in numerical format. The importance of wording has also been confirmed in our recent studies: when low-risk options were framed as potential monetary losses, high anxious participants showed no preference toward these kinds of options (Xu et al., 2013; Gu et al., 2017).

Regarding ambiguous decision-making, some studies suggest that high anxious individuals show different behavioral preferences compared to their low anxious counterparts, but other studies disagree (e.g., Wray and Stone, 2005; Gu et al., 2010a,b). During ambiguous decision-making, when people are allowed to make multiple choices under the same rules, they rely on trial-and-error reward learning to explore the underlying outcome probabilities (Dayan and Niv, 2008; Peterson et al., 2011). It is also debated whether anxiety affects reward learning ability: Dickstein et al. (2010) and Browning et al. (2015) did not find any association between anxiety level and overall reward learning rate. However, according to one of our recent studies, both behavioral response and outcome evaluation are modulated by anxiety during reward learning (Jiang et al., 2018). In a word, whether high levels of anxiety are related to altered behavioral performance in risk and/or ambiguous decision-making is still largely undetermined.

In our opinion, gender difference might have contributed to the heterogeneous findings in the literature. As pointed out by Schiebinger (2014), the significance of gender for scientific research should be highlighted (see also Eliot, 2011). It has long been established in laboratory studies that men and women participants differ in various cognitive and emotional processes (e.g., Kemp et al., 2004; Marumo et al., 2009; Scheele et al., 2014; Weisenbach et al., 2014; Mieth et al., 2017). Gender difference also manifests in decision-making; for instance, men show heightened levels of reward drive compared to women (Loxton et al., 2008). Regarding risk decision-making, men are more likely to take risks than women generally (Lauriola and Levin, 2001; Loewenstein et al., 2001). Regarding ambiguous decision-making, men perform better than women and show a more goal-directed behavioral pattern in the classic Iowa Gambling Task (IGT: see below for details; Overman et al., 2004; Van den Bos et al. 2007, 2009). The potential neural underpinnings of these gender differences have been discovered. For instance, men and women show different brain activation patterns (indicated by the levels of hemodynamic and electrophysiological responses) during decision-making (Kamarajan et al., 2008, 2009; Lighthall et al., 2012). Importantly, an interaction between anxiety and gender has also been observed. Although gender is not often considered as a potential confounding factor in the research on anxiety and decision-making, de Visser et al. (2010) found that both high anxious and medium anxious men showed impaired IGT performance, whereas in women only high anxious participants showed impaired performance. Their findings indicate the necessity to take gender into account when investigating the relationship between anxiety and decision-making.

Accordingly, the current study explored the possibility that the influence of anxiety on decision-making is modulated by gender. Both risk decision-making and ambiguous decision-making were examined. Following the major interest of our previous research (e.g., Wu et al., 2013; Luo et al., 2014; Wang et al., 2017), we focused on individual trait anxiety level, which refers to the disposition to experience anxiety-relevant feelings, rather than the transient level of anxiety state (Spielberger et al., 1983; Bekker et al., 2003). The Game of Dice Task (GDT) was used to measure risk decision-making (Brand et al., 2005). Like the IGT, the GDT has also been frequently employed in the laboratory environment (e.g., Brand et al., 2006). However, limited research has been done to investigate the role of emotion in the GDT, except for some notable clinical studies: Patients with attention-deficit/hyperactivity disorder (ADHD) made more risky choices than the controls in the GDT (Drechsler et al., 2008), but those with obsessive–compulsive disorder (OCD) showed unimpaired GDT performance (Starcke et al., 2010). Meanwhile, the IGT was used to measure ambiguous decision-making (Bechara et al., 1994). As introduced above, the IGT performance could be modulated by both anxiety and gender. Using both tasks in the same sample, Zhang et al. (2015) concluded that anxiety has an effect on ambiguous decision-making (IGT), but not risk decision-making (GDT). Nonetheless, Zhang et al. (2015) did not consider the potential role of gender. We predicted that the IGT would represent an interaction between anxiety and gender, similar with the findings from de Visser et al. (2010). Regarding the GDT, however, no prior hypothesis has been made due to the absence of prior studies.

## Materials and Methods

### Participants

Three hundred and sixty-seven undergraduate students (171 men, 196 women) participated in a mass screening using the Chinese version of the Trait form of Spielberger’s State-Trait Anxiety Inventory (STAI-T: Spielberger et al., 1983; Shek, 1993), which has demonstrated good internal consistency and discriminant validity (Barnes et al., 2002). The mean STAI-T score of the whole sample (42.78 ± 7.71) was similar with the standardized norm of STAI-T among Chinese undergraduate students (43.31 ± 9.20: see Li and Qian, 1995). Individuals who scored 1 standard deviation above or below this mean score were categorized as high or low in trait anxiety, while all other individuals were categorized as medium in trait anxiety (following the categorization method of de Visser et al., 2010). After the categorization stage, we randomly recruited participants from the original sample of 367 people. Using G^∗^Power (version 3.1.7^1^; Faul et al., 2007), we found in a priori analysis that a total sample size of 126 would ensure 80% statistical power in case of small-to-medium effect sizes, which is consistent with the suggestion from Vazire (2016).

A total of 135 students (mean age = 20.52 ± 1.25) accepted our invitation and finished the formal experiment. Consequently, there were 21 men and 20 women in the high trait anxiety (HTA) group, 24 men and 29 women in the medium trait anxiety (MTA) group, and 16 men and 25 women in the low trait anxiety (LTA) group. All of them had normal visual acuity and none had medical history or psychological disorders according to self-report. All participants gave their written informed consent prior to the experiment. The experimental protocol was approved by the local ethics committee (Jiangxi Normal University).

### Experimental Paradigm: IGT

**FIGURE 1:**
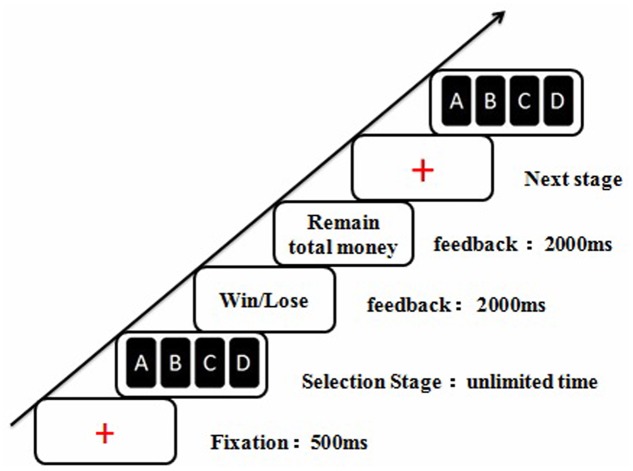
An exemplar trial in the Iowa Gambling Task (IGT). ms, milliseconds.

We used a computerized version of the IGT to measure decision preference under ambiguity (Bechara et al., 1994). At the beginning of the task, participants were given a loan of 2000 points. In each trial of the task (see **Figure 1**), participants first saw a fixation point for 500 ms, and then chose one card from four decks of cards (A, B, C, and D), which would result in win or loss. Consistent with the design of the classic IGT, decks A and B produced large immediate gains (an average gain of 100 points for each win), but they were long-term disadvantageous decks (-250 points pre 10 cards); in contrast, decks C and D produced small immediate gains (an average gain of 50 points for each win), but they were long-term advantageous decks (Bechara et al., 1994). The four decks remained on the screen until participants made a selection, followed by the outcome feedback (a Chinese word “win/lose”) of the current trial for 2000 ms. After that, the updated total score amount also appeared for another 2000 ms, indicating the end of the current trial. There were 100 trials in the task.

Participants had no prior knowledge about the potential payoff and winning probability associated with each deck. In other words, the IGT was an ambiguous decision-making task.

### Experimental Paradigm: GDT

**FIGURE 2:**
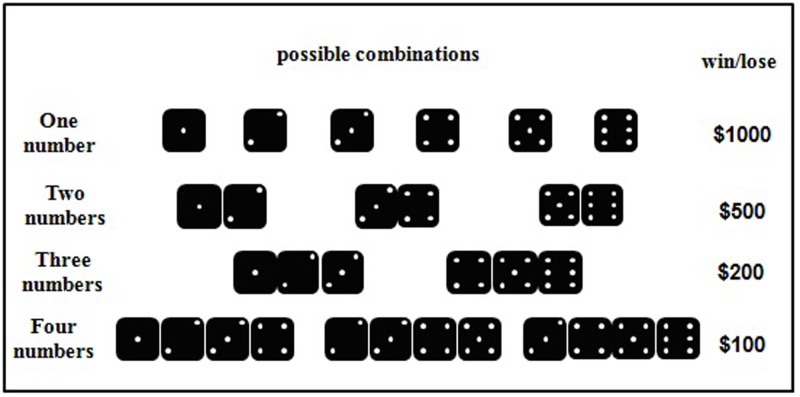
The possible die combinations associated with each option in the Game of Dice (GDT) task.

We used a computerized version of the GDT to measure decision preference under risk (Brand et al., 2005). At the beginning of the task, participants were also given a loan of 2000 points. In each trial of the task (see **Figure 2**), participants first saw a fixation point for 500 ms, and then chose between four options (1, 2, 3, 4), which would result in win or loss. The GDT required participants to roll a virtual die, and the four options represent the number of dice combinations for participants to bet on. Each option is associated with defined payoff and winning probability: there was a probability of 1:6 to win 1000 points by choosing the option “1,” 2:6 to win 500 points by choosing “2,” 3:6 to win 200 points by choosing “3,” and 4:6 to win 100 points by choosing “4.” Accordingly, the options “1” and “2” were defined as high-risk choices, and the options “3” and “4” were defined as low-risk choices. The four options remained on the screen until participants made a selection, following by the result of die throws of the current trial for 2000 ms. After that, the updated total score amount also appeared for another 2000 ms, indicating the end of the current trial. There were 18 trials in the task.

Because participants had knowledge about the potential payoff and winning probability associated with each option, the GDT was defined as a risk decision-making task.

### Experimental Design and Procedure

The study employed a 3 (Group: HTA/MTA/LTA) × 2 (Gender: men/women) random experimental design. Within 1 week after the mass screening (see above), participants were invited to the laboratory to finish the formal experiment. The operating procedures of the IGT and GDT were explained in detail, and all participants confirmed that they fully understood the two tasks. They also finished a short practice to be familiar with each task. Participants were encouraged to maximize the amount of total scores that they could get from the tasks.

All the participants finished the two tasks in sequence, such that half of the participants were randomly selected to finish the IGT first, and the other half finished the GDT first. There was a break of 5 min for rest between the two tasks. The whole experiment continued for about 30 min. The stimuli were presented and behavioral responses were collected using E-Prime (Version 1.1, PST, Inc., Pittsburgh, PA, United States).

At the end of the experiment, the participants were debriefed. Because the university did not encourage using monetary remuneration, each participant received a small gift which valued 30–80 Chinese RMB depending on their task performance.

### Statistics

Regarding the IGT, a net score (CD – AB) was calculated to estimate individual task performance, that is, the difference in the numbers of advantageous choices (choosing C or D) and disadvantageous choices (choosing A or B). Consistent with previous studies using the IGT (e.g., Zhang et al., 2015), the 100 trials were divided into five equal blocks and the net score of each block was calculated separately, so as to investigate whether decision behavior changed during the task. The block factor was considered as a within-subject factor in statistical analysis.

Regarding the GDT, a net score was calculated by subtracting the number of high-risk choices (1 or 2) from the number of low-risk choices (3 or 4), such that a larger net score indicate a stronger tendency to avoid risk (Brand et al., 2005).

The significance level was set at 0.05. Significant interactions were analyzed using simple effects models. Greenhouse–Geisser correction for the analysis of variance (ANOVA) tests was used whenever appropriate. *Post hoc* testing of significant main effects and multiple comparisons were conducted using the Bonferroni method. Partial eta-squared ($\eta_{p}^{2}$) was reported to demonstrate the effect size of the analysis of variance (ANOVA) tests, where 0.05 represents a small effect, 0.1 represents a medium effect, and 0.2 represents a large effect (Cohen, 1973). Statistical analyses were conducted with SPSS 18.0 (SPSS, Chicago, IL, United States). Descriptive data are presented as mean ± standard deviation.

## Results

### STAI-T Score

The STAI-T score of each group was: HTA men: 53.95 ± 3.90; HTA women: 52.70 ± 5.72; MTA men: 42.88 ± 3.26; MTA women: 43.07 ± 4.40; LTA men: 33.38 ± 4.10; and LTA women: 32.48 ± 3.27. The STAI-T score of the MTA group was similar with the standardized norm of STAI-T among Chinese undergraduate students (43.31 ± 9.20; see above).

A 3 (Group: HTA/MTA/LTA) × 2 (Gender: men/women) ANOVA test with Bonferroni correction was performed to examine the STAI-T score. There was a significant main effect of Group [*F*(2,132) = 241.62, *p* < 0.001, $\eta_{p}^{2}$ = 0.79], indicating that the trait anxiety level was different between groups. However, there was no significant main effect of Gender [*F*(1,133) = 0.80, *p* = 0.37, $\eta_{p}^{2}$ = 0.01]. Likewise, the Gender by Group interaction was not significant [*F*(5,129) = 0.39, *p* = 0.68, $\eta_{p}^{2}$ = 0.01].

### IGT Performance

A 3-way repeated-measures ANOVA with two between-subjects factors (Group: three levels; Gender: two levels) and one within-subjects factor (Block: five levels) was used to examine the IGT net score.

**FIGURE 3:**
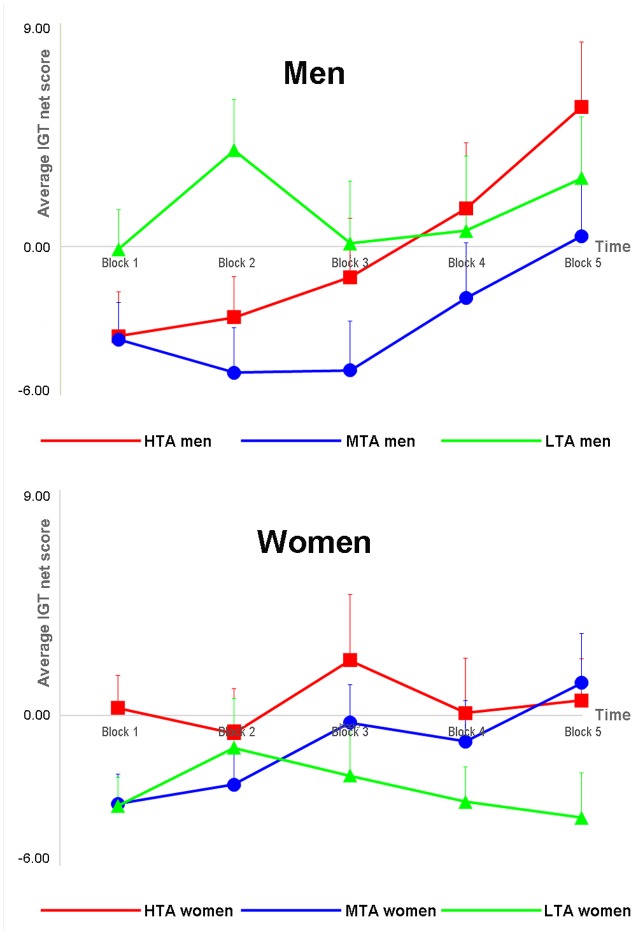
The average net score (calculated by subtracting the number of disadvantageous choices from that of advantageous choices) in each block (i.e., 20 trials) of the Iowa Gambling Task (IGT) for male (upper) and female (lower) participants.

The main effects of Group [*F*(2,129) = 1.420, *p* = 0.245] and Gender [*F*(1,129) = 0.293, *p* = 0.589] were insignificant. However, the results showed a main effect of Block [*F*(3.41, 440.40) = 4.768, *p* = 0.002, $\eta_{p}^{2}$ = 0.036]; the net score gradually increased over time (see **Figure 3**).

There was a noticeable Group × Block interaction [*F*(6.83,440.40) = 1.995, uncorrected *p* = 0.045, corrected *p* = 0.056, $\eta_{p}^{2}$ = 0.030]; LTA participants showed better performance than MTA participants in Block 2 (*p* = 0.003), but no other significant difference was detected (*ps* > 0.05). Meanwhile, there was a significant Gender × Block interaction [*F*(3.41,440.40) = 3.073, *p* = 0.022, $\eta_{p}^{2}$ = 0.023]; men showed better performance than women in Block 5 (*p* = 0.044), but not in other blocks (*p*s > 0.05). There was also a noticeable Group × Gender interaction [*F*(2,129) = 2.486, *p* = 0.087, $\eta_{p}^{2}$ = 0.037], though it did not reach a statistically significant level; pairwise comparisons revealed that men performed better than women in LTA groups (*p* = 0.047), but not in other groups (*p*s > 0.05). Finally, the Group × Gender × Block interaction was insignificant [*F*(6.83,440.40) = 0.913, *p* = 0.495].

We also conducted a linear contrast analysis, which showed that the linear contrast of the main effect of block was significant [*F*(1,129) = 10.645, *p* = 0.001], indicating that the IGT performance gradually became better across blocks (see **Figure 3**). The linear contrast of the Group × Block interaction [*F*(2,129) = 2.725, *p* = 0.069] was noticeable, though not significant; the linear trend was significant in the HTA [*F*(1,40) = 6.394, *p* = 0.016] and MTA [*F*(1,52) = 9.621, *p* = 0.003] groups, but was insignificant in the LTA group [*F*(1,40) = 0.061, *p* = 0.805]. The linear contrast of the Gender × Block interaction [*F*(1,129) = 3.621, *p* = 0.059] was also noticeable, though not significant; the linear trend was significant in men [*F*(1,60) = 10.583, *p* = 0.002], but not in women [*F*(1,73) = 1.808, *p* = 0.183]. Finally, the linear contrast of the Group × Gender × Block interaction was insignificant [*F*(2,129) = 1.888, *p* = 0.156].

### GDT Performance

A 3 (Group: 3 levels) × 2 (Gender: 2 levels) ANOVA was performed to examine the GDT net score (see **Table 1** for details). There was a noticeable main effect of Group [*F*(2,127) = 3.009, *p* = 0.053, $\eta_{p}^{2}$ = 0.045], though not statistically significant; however, pairwise comparisons showed no difference between groups (*p*s > 0.05), although LTA participants manifested a stronger tendency to make more risky decisions than MTA participants (3.35 ± 1.82 vs. 8.82 ± 1.57; *p* = 0.073). In contrast, no difference was detected when comparing the HTA (4.56 ± 1.76) and MTA groups (*p* = 0.219), or the HTA and LTA groups (*p* = 1.000). The main effect of Gender [*F*(1,127) = 1.010, *p* = 0.317] and the Group × Gender interaction [*F*(2,127) = 0.172, *p* = 0.842] were insignificant.

**Table 1:** Net score (mean ± standard deviation) in the Game of Dice Task (GDT) per group.

	High trait anxiety (HTA)		Medium trait anxiety (MTA)		Low trait anxiety (LTA)	
	Men	Women	Men	Women	Men	Women
GDT net score	3.62 ± 14.22	5.50 ± 10.50	8.50 ± 10.86	9.14 ± 7.93	1.62 ± 13.73	5.08 ± 10.91

## Discussion

In this study, we examined the potential interaction between anxiety and gender by using both the IGT and GDT in the sample. Regarding the IGT, the average net score gradually increased across different blocks, which was consistent with previous studies using the IGT (Bechara et al., 2005). This result confirms that the participants were actively engaged in the task. We did not detect the main effect of anxiety, although there was an interaction of Group by Block, indicating that LTA groups performed better than MTA groups in Block 2. We admit that the theoretical significance of this finding is unclear to us. It is possible that LTA participants learned the winning rules faster than MTA participants during certain stages in the IGT. Nevertheless, the relatively small sample size (41–53 people per group) in this study might also be an issue, and follow-up verification would be necessary (but see Zhang et al., 2015).

The linear contrast analysis indicated that the linear trend was significant in both the HTA and MTA groups, but not in the LTA group. One possible explanation of this phenomenon is that the task performance of the LTA group reached its peak at the early stage (see **Figure 3**). Meanwhile, the linear trend was significant in men but not in women, which is in line with the classic gender effect of the IGT in previous studies (see the Introduction).

Most importantly, there was a noticeable (though insignificant) Group by Gender interaction, indicating that the influence of anxiety on IGT choices was indeed modulated by gender. Specifically, men participants outperformed women participants only when the level of anxiety was low. As described in the Introduction section, it is well established that there is a significant gender difference in the IGT; generally, men tended to choose the options with long-term benefit more than women (Bolla et al., 2004; Reavis and Overman, 2011). Summarizing 158 studies, van den Bos et al. (2013) concluded two reasons for this gender difference: first, women tend to focus on the frequency of winning and losing while men focus more on the long-term benefits; second, women may be more sensitive to occasional losses in the long-term advantageous decks than men. The current study, however, reveals that with heightened levels of anxiety, the gender difference in the IGT disappears. One possible explanation for this phenomenon is that anxiety amplifies the subjective feelings to short-term losses in both men and women, thus making it more difficult to find the advantageous decks in the IGT. Our previous event-relate potential (ERP) studies which focused on the impact of anxiety on outcome evaluation, supports this explanation (Gu et al., 2010a,b; Luo et al., 2014; see also Takács et al., 2015).

Meanwhile, there was a noticeable main effect of anxiety in the GDT, such that LTA groups tended to be more risk-seeking that MTA groups. This finding highlights the importance to recruit a medium anxiety group, as some previous studies which only compared HTA and LTA individuals did not find any effect of anxiety on risk preference (Luo et al., 2014; Xia et al., 2017). According to Zhang et al. (2015), the effect of trait anxiety manifested on the IGT, but not on the GDT; they accordingly proposed that anxiety selectively affects ambiguous decision-making but not risk decision-making. The current results, however, suggest that the effect of anxiety on the GDT is observable. Though no conclusion could be drawn here about this inconsistency, it is worth noting that the anxiety levels of the MTA and LTA groups (37.84 and 26.23, respectively) in the study of Zhang et al. (2015) deviated from our sample. Therefore, the “MTA” and “LTA” groups in their study might actually reflect different populations from those in the current study, regardless of the same labels. To explain the finding that LTA participants made more risky choices than MTA (but not HTA) participants, one possibility is that anxiety affects risk preference in two different aspects. On the cognitive level, anxiety may strengthen an attentional bias toward potential losses (Hartley and Phelps, 2012); meanwhile, on the physiological level, anxiety leads to stronger autonomic arousal responses. Importantly, these two aspects might counteract each other, as individuals with a heightened arousal level are actually more likely to be risk-seeking (Mano, 1992). Consequently, HTA participants (with highest arousal level in the sample) showed no difference with LTA participants in risk preference.

To sum up, this study found that the influence of anxiety on decision-making could be modulated by gender in the ambiguous condition, but not in the risk condition. Considering that, the heterogeneity in previous studies might be partly due to the varied gender ratio of different samples. Thus, it would be necessary to conduct gender analysis for the research on anxiety and decision-making, and maybe for behavioral economics more broadly. Also, the neural mechanisms of the current findings would be interesting for further research to investigate. It has long been acknowledged that risk decision-making and ambiguous decision-making have distinct neural underpinnings (Krain et al., 2006). According to Levy et al. (2010), the posterior cingulate cortex (PCC), superior temporal sulcus (STS), and amygdala might be uniquely activated under the conditions of ambiguity but not risk. We suggest follow-up studies to examine whether an interaction of anxiety and gender would manifest in these brain regions.

## Ethics Statement

This study has been approved and performed in accordance with the guidelines for the ethics committees of Jiangxi Normal University. This study has not been submitted to any other Journals. We have followed the guidance of the APA requirements of human subjects.

## Author Contributions

FZ and LX equally contributed to the design of the study. LX prepared the experimental materials, collected the data, analyzed the results and wrote the paper. FZ contributed to experimental materials, participated to part of the data analysis, programmed the experiment, collected the data. All authors reviewed the manuscript.

## Conflict of Interest Statement

The authors declare that the research was conducted in the absence of any commercial or financial relationships that could be construed as a potential conflict of interest.

## Footnotes

1 http://gpower.hhu.de/
